# A Two-Step Neural Dialog State Tracker for Task-Oriented Dialog Processing

**DOI:** 10.1155/2018/5798684

**Published:** 2018-10-18

**Authors:** A-Yeong Kim, Hyun-Je Song, Seong-Bae Park

**Affiliations:** ^1^School of Computer Science and Engineering, Kyungpook National University, 80 Daehakro, Buk-gu 41566, Daegu, Republic of Korea; ^2^Naver Search, Naver Corporation, 6 Buljeong-ro, Bundang-gu, Seongnam 13561, Gyeonggi, Republic of Korea; ^3^Department of Computer Science and Engineering, Kyung Hee University, 1732 Deogyeong-daero, Giheung-gu, Yongin 17104, Gyeonggi, Republic of Korea

## Abstract

Dialog state tracking in a spoken dialog system is the task that tracks the flow of a dialog and identifies accurately what a user wants from the utterance. Since the success of a dialog is influenced by the ability of the system to catch the requirements of the user, accurate state tracking is important for spoken dialog systems. This paper proposes a two-step neural dialog state tracker which is composed of an informativeness classifier and a neural tracker. The informativeness classifier which is implemented by a CNN first filters out noninformative utterances in a dialog. Then, the neural tracker estimates dialog states from the remaining informative utterances. The tracker adopts the attention mechanism and the hierarchical softmax for its performance and fast training. To prove the effectiveness of the proposed model, we do experiments on dialog state tracking in the human-human task-oriented dialogs with the standard DSTC4 data set. Our experimental results prove the effectiveness of the proposed model by showing that the proposed model outperforms the neural trackers without the informativeness classifier, the attention mechanism, or the hierarchical softmax.

## 1. Introduction

Dialog systems for a task-oriented dialog facilitate the interactions between the systems and their users by a natural language to achieve the requirements from the users. Thus, the task-oriented dialog systems carry out several tasks such as identifying the user intention from a user utterance, tracking a dialog flow, and planning a dialog strategy to fulfill the requirements. Furthermore, they generate a natural language response for the user. Since the ability of a task-oriented dialog system to catch user requirements and to recognize a dialog flow influences its success heavily, it is essential to track what has happened and estimate the needs of a user.

The dialog state tracker in a task-oriented dialog system estimates the state of a conversation from a user utterance. Then, the dialog system plans a dialog strategy using the state to reach a dialog goal that fulfills the user requirements. Usually, the tracker has a slot-filling architecture which is based on predefined semantic slots to translate user utterances into a semantic representation [1]. Thus, a dialog state is expressed as a set of slot-attribute pairs. As a result, the objective of the dialog state tracker is to estimate a true slot and its attributes from a user utterance.

The success of a task-oriented dialog depends greatly on how quickly and precisely the dialog states are caught from user utterances [2, 3]. There have been a number of studies that make a dialog state tracker more precise and quicker [4, 5]. However, the previous studies have two kinds of problems in common. The first problem is that the previous studies assume that every utterance delivers slot-attribute pairs. As a result, they try to estimate slot-attribute pairs for all user utterances. However, some utterances do not carry any information about true slot and true attributes of the slot. For instance, “Hi. My name is Arlyn”is an utterance for responding to a system utterance, and “Hello” is an opening speech but has no information about dialog state. Therefore, such noninformative utterances should be filtered out before estimating a dialog state from an utterance.

Another problem is that task-oriented dialogs cover a large state space. As a result, many slot-attribute pairs occur rarely in the training data even if they become a dialog state. The dialog state tracking can be formulated as a classification task in which the slot-attribute pairs are classes. However, the classifiers based on traditional machine-learning methods including the neural networks with the softmax do not work well in this task due to a great number of classes and infrequent classes. To resolve this problem, the dialog state tracker should not consider only numerous number of classes, but should be able to predict also rare classes.

This paper proposes a two-step neural tracker that solves the problems. The first step of the proposed tracker filters out the noninformative utterances. Filtering out noninformative utterances can be regarded as a binary classification which determines whether a given utterance has meaningful words for tracking dialog states or not. Since convolutional neural networks (CNNs) show good performance in sentence classification [6, 7], this paper adopts a CNN to filter out noninformative utterances. The second step of the proposed tracker determines actual dialog states by using an attention-based recurrent neural network (RNN) with the hierarchical softmax function. The reason why RNN is adopted in the second step is that it is effective in classifying dynamic sequences with complex behaviors [8]. This RNN-based dialog state tracker employs the attention mechanism and the hierarchical softmax, since the attention mechanism shows good performances in various NLP tasks [9] and helps a model focus on valuable words in an utterance. In addition, the adoption of the hierarchical softmax [10, 11] reduces the computational complexity in training of the proposed model. Dialogs are usually described with a large vocabulary, and every word in the vocabulary can be an attribute or a class in dialog state tracking. Since the hierarchical softmax can deal with a large number of classes efficiently, the proposed tracker can make good estimations of dialog states for infrequent classes as well as for frequent classes.

The rest of this paper is organized as follows.  Section 2 discusses the previous studies on dialog state tracking.  Section 3 proposes the two-step neural dialog state tracker and Section 4 describes the details of the two-step neural dialog state tracker. An evaluation of the proposed model is given in Section 5. Finally, Section 6 concludes this paper.

## 2. Related Work

Conventional dialog systems use handcrafted heuristics to update proper dialog states from the output of a natural language understanding module [12, 13]. For instance, Zue et al. proposed handcrafted update rules to manage dialog states in a weather information system [14], and Larsson and Traum adopted handwritten rules to track information states which represent the necessary information for tracking a dialog flow [15]. On the other hand, Sun et al. proposed a rule-based method to compute the confidence scores of the N-best candidates generated by their spoken language understanding module [16]. However, these rules are not derived automatically from real dialog data so that they require careful tuning and delicate designing efforts. Due to such characteristics of rule-based methods, these methods often result in inaccuracy of determining dialog states.

As a solution to handcrafted rules, statistical methods have been applied to dialog state tracking [17–19]. Bohus and Rudnicky used a logistic regression to provide an initial assessment of the reliability of the information obtained from a user [17]. To achieve high tracking performance, they adopted some features such as the prior likelihoods of dialog state slots. Thomson and Young represented dialog state tracking with a Bayesian network and proposed a belief update algorithm for resolving the tractability of inference in the Bayesian network [18]. Their experimental results prove that the model can not only deal with a very large number of slots, but also it achieves significant improvement over the systems with handcrafted rules. Ma et al. proposed a model with two trackers for a location-based dialog system [19]. One tracker is designed with a Bayesian network to track nonlandmark concepts such as a company or a street, while the other is a kernel-based tracker that deals with landmark concepts in a dialog corpus. However, these studies share a common problem that they have to enumerate all possible dialog states which is computationally very expensive.

Due to increasing interests on dialog state tracking, dialog state tracking challenges (DSTC) have been held for the last several years. DSTC provided a fully labeled data set and an evaluation framework for benchmark tasks. This data set has evolved from human-machine dialogs to human-human task-oriented dialogs and from a single domain to multiple domains. For instance, a person requests bus information to a machine in the DSTC1. Thus, the machine leads dialogs in DSTC1. On the other hand, a person requests tour information to a (human) tour guide in DSTC4. Since the DSTC data set is used in most recent studies [20, 21], it is now regarded as a standard data set for dialog state tracking.

One notable aspect of DSTC is that the number of studies which adopt a neural network for dialog state tracking is increasing explosively. Henderson et al. first applied a neural network to dialog state tracking [2]. Their study is meaningful in that it is the first try of using a neural network for dialog state tracking, but their method still relies much on handcrafted features. After that, they proposed another neural network-based model without any handcrafted features or explicit semantic representation [8]. This model extracts its feature representation automatically from the N-best list of speech recognition and machine acts. Perez and Liu used the memory network for easy handling of dialog history as well as identifying user request [22]. They generated question-answer pairs manually to estimate what slot-attribute pairs are actually needed for a subdialog. However, the main problem of these studies is that they are based on the assumption that each component of a dialog system is developed independently. As a result, the problem of error propagation still remains.

The most recent neural approach to dialog state tracking accepts raw user utterances directly rather than analyzing the utterances through a natural language understanding module. For instance, Yoshino et al. proposed a neural tracker based on the long short-term memory (LSTM) [20]. The input to this LSTM is raw dialog utterances of a user. On the other hand, Shi et al. used a CNN to focus on tracking general information in a subdialog segment [21]. To deal with multitopic dialogs, their CNN is composed of general and topic-specific filters that share topic information with each other. They showed meaningful results in their experiments for multiple topics, but their model tends to miss the right timing when the tracker should generate its output for some specific slots.

## 3. Two-Step Neural Dialog State Tracker

**Figure 1:**
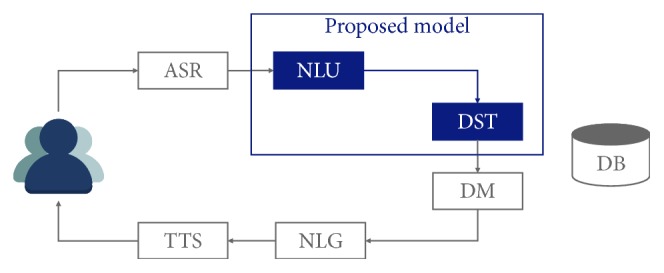
The general structure of traditional task-oriented dialog systems. The proposed dialog tracker corresponds to both NLU and DST of the traditional systems.

Figure 1 shows the general structure of traditional task-oriented dialog systems. The systems usually consist of six submodules which are ASR (automatic speech recognition), NLU (natural language understanding), DST (dialog state tracking), DM (dialog manager), NLG (natural language generation), and TTS (text-to-speech). Since they are pipelined, the errors of one module are easily propagated to subsequent modules. Thus, it is of importance to reduce the number of modules as well as to minimize the errors of each module. This paper proposes a neural dialog state tracker in an end-to-end style. That is, the tracker does not use any output from NLU. Instead, it takes the result of ASR as its input directly and returns a relevant dialog state as its output. Therefore, it actually substitutes two components of NLU and DST in this figure.

**Figure 2:**
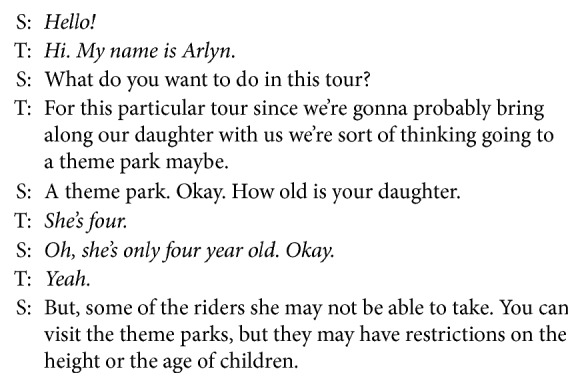
An example dialog about a tour booking with some dialog state independent utterances. S in this example is a system, and T is a tourist.

The task-oriented dialogs usually proceed in four steps of *opening*, *asking*, *offering*, and *ending*. There could be many user utterances in each step that are not directly related with dialog states. Especially, the utterances in the opening and the ending steps deliver no information about dialog states, since they are usually greetings and closing remarks. However, *asking* and *offering* steps also can have such utterances. For instance, let us consider an example dialog for tour booking in Figure 2. While the bold utterances deliver the type of a tour place and restriction information, the italic utterances have nothing to do with dialog state. Note that such noninformative utterances can appear between informative utterances. These noninformative utterances result in the performance degradation of a dialog state tracker if they are given as an input to the tracker. Thus, they should be filtered out before tracking dialog states.

**Figure 3:**
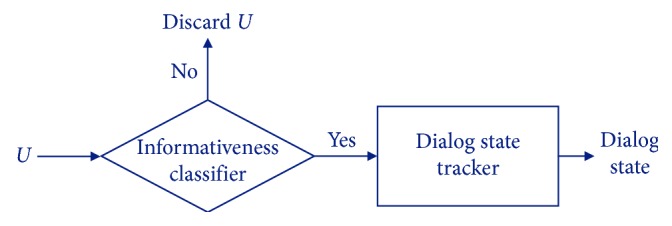
The overall structure of the proposed two-step neural tracker.

To filter out noninformative utterances before determining actual dialog states, the proposed tracker has two steps as shown in Figure 3. In the first step, the informativeness classifier determines whether a user utterance *U* is informative or not. If it decides that *U* is noninformative, then *U* is discarded. Otherwise, the dialog state of *U* is then classified by the actual dialog state tracker. Since both the informativeness classifier and the dialog state tracker are implemented with neural networks in this paper, each user utterance is represented to a sequence of word vectors by Word2Vec [23].

## 4. Implementation of Two-Step Tracker

### 4.1. Informativeness Classifier

Convolutional neural networks have been reported to be outstanding in many NLP tasks including sentence classification [6, 24]. Thus, a CNN is adopted for the informativeness classifier in Figure 3. It accepts the numerical utterance vector as its input. Assume that the user utterance *U* consists of *n* words. That is, *U*={*x*_1_, *x*_2_,…, *x*_*n*_}, where *x*_*i*_ is the *i* − th word in *U*. If *x*_*i*_ is represented as a *k*-dimensional vector **x**_**i**_ by Word2Vec, then the utterance vector **U**_1:*n*_ becomes

(1) $$\begin{array}{c} U_{1:n}=x_{1}\;⊕\;x_{2}\;⊕\;\cdots \;⊕\;x_{n}, \end{array}$$

where ⊕ is the concatenation operator.

**Figure 4:**
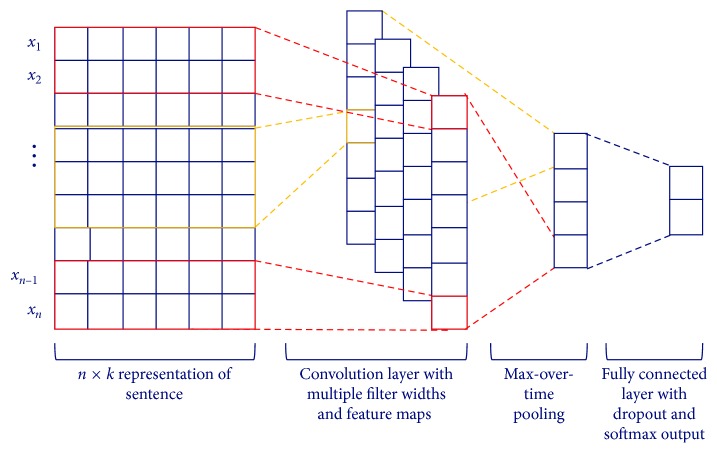
The structure of the informativeness classifier.

The informativeness classifier determines whether the utterance delivers some information about dialog states or not.  Figure 4 depicts the structure of the informativeness classifier. The classifier is a CNN with a convolution layer, a max pooling layer, a regularization layer, and a softmax layer. The convolution layer makes multiple feature maps with multiple filters, where each filter **f**_*w*_ ∈ ℝ^*wk*^ is applied to a window of *w* words to produce a new feature for the next layer. That is, a new feature *c*_*iw*_ is derived from a filter **f**_*w*_ and a window of words by

(2) $$\begin{array}{c} c_{iw}=t\left(f_{w}\cdot U_{i:i+w-1}+b\right), \end{array}$$

where *t* is a nonlinear function such as the hyperbolic tangent and *b* is a bias term. This filter is applied to every window of words in *U* to produce a feature map with a window size *w*:

(3) $$\begin{array}{c} c_{w}=\left[c_{1w},c_{2w},\ldots ,c_{n-w+1,w}\right]. \end{array}$$

Multiple filters with various window sizes of words are used to obtain multiple feature maps.

The max pooling layer captures the most important feature for each feature map. When a max pooling operation is applied to a feature map **c**_*w*_, the maximum value of the map, ${\hat{c}}_{w}=\max\left(c_{w}\right)$, is obtained as a feature corresponding to the particular filter **f**_*w*_. This pooling scheme deals with variable-length inputs and produces fixed-length outputs for the subsequent layers. A dropout is used for the regularization of this CNN before the outputs of max pooling layer are passed to a fully connected softmax layer whose output is the probability distribution over informativeness labels.

### 4.2. Neural Dialog State Tracker

**Figure 5:**
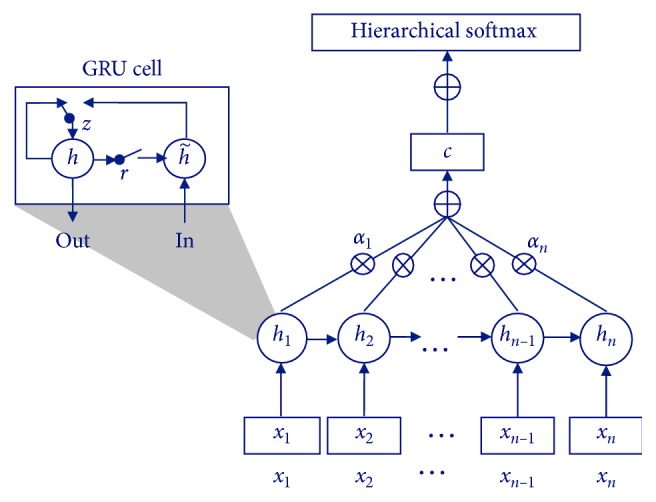
The structure of the proposed dialog state tracker with attention and hierarchical softmax.

Figure 5 shows the structure of the proposed dialog state tracker. This tracker consists of a RNN-based encoder and an output decoder, where the decoder is composed of an attention layer, a linear layer, and a softmax layer. The gated recurrent unit (GRU) is used for the encoder, since it is widely used in recurrent neural networks to encode a sentence or an utterance. Each GRU cell takes an input vector **x**_**i**_, and calculates the hidden state *h*_*i*_ using a reset gate *r*_*i*_, an update gate *z*_*i*_, and weight matrices *W* and *M* for the two gates. The variables are actually computed by

(4) $$\begin{array}{c} h_{i}=\left(1-z_{i}\right)h_{i-1}+z_{i}{\tilde{h}}_{i},\\ z_{i}=\sigma \left(W_{z}x_{i}+M_{z}h_{i-1}\right),\\ r_{i}=\sigma \left(W_{r}x_{i}+M_{r}h_{i-1}\right), \end{array}$$

where the hidden state *h*_*i*_ of a GRU cell at time *i* is a linear interpolation between the previous hidden state *h*_*i*−1_ and the candidate hidden state ${\tilde{h}}_{i}$. The update gate *z*_*i*_ decides how much the unit updates its content, and *r*_*i*_ is a reset gate that determines whether the GRU cell forgets the previously computed state or not. Then, ${\tilde{h}}_{i}$, the candidate hidden state, is updated using the current input, the reset gate, and the previous hidden state. That is,

(5) $$\begin{array}{c} {\tilde{h}}_{i}=\tanh\left(Wx_{i}+M\left(r_{i}\;⊙\;h_{i-1}\right)\right), \end{array}$$

where ⊙ is an element-wise multiplication.

The slot and its attribute for a dialog state are estimated by the decoder. The input for the decoder is an encoded representation *h*_1:*n*_ of the utterance. Since it is important in estimating slots to focus on some words containing information about a dialog state, an attention mechanism is adopted in this architecture. Bahdanau et al. showed that a basic encoder-decoder architecture with an attention mechanism outperforms the architecture without attention at machine translation tasks [9]. This implies that the attention mechanism can tell roughly the decoder to which words the decoder has to pay attention in translating a sentence. In dialog state tracking, the decoder is able to catch salient words from an input utterance by adopting an attention mechanism.

The attention mechanism used in the proposed model first calculates the normalized alignment scores that refer to how closely the words from *x*_*i*_ to *x*_*j*_ are related. The alignment score of each hidden layer output is calculated by

(6) $$\begin{array}{c} e_{ij}=a\left(h_{i},h_{j}\right), \end{array}$$

where *a* is an alignment model that is implemented by a feedforward network. Before computing the salience vector *s*_*i*_, the alignment score is normalized by

(7) $$\begin{array}{c} \alpha_{ij}=\frac{\exp\left(e_{ij}\right)}{{\sum_{l=1}^{n}\exp\left(e_{il}\right)}^{}}. \end{array}$$

Then, the salient vector *s*_*i*_ that indicates the significance of the output of a hidden layer is calculated as a weighted sum of the hidden layer output and the normalized alignment scores. That is,

(8) $$\begin{array}{c} s_{i}=\overset{n}{\underset{j=1}{\sum }}\alpha_{ij}h_{i}. \end{array}$$

After all salience vectors are obtained, they are averaged into a single vector as

(9) $$\begin{array}{c} s=\frac{1}{n}\overset{n}{\underset{j=1}{\sum }}s_{i}. \end{array}$$

Then, the dialog state is estimated from **s**. That is, **s** is inputted to the softmax layer with a hierarchical softmax. The hierarchical softmax reduces the computation complexity by representing all classes as a binary tree [11]. A leaf node of the tree represents a class, and an intermediate node contains the common representation of its child nodes. Thus, unlike the flat softmax in which every class is processed independently, the hierarchical softmax makes a class be complemented by other classes through the intermediate nodes [25]. As a result, this hierarchical softmax gets able to estimate infrequent classes. In general, a dialog has a large collection of vocabulary, and every word in the vocabulary becomes a class in dialog state tracking. Thus, due to a great number of classes and infrequent classes in dialog state tracking, it is difficult for a tracker to estimate the dialog state. However, by adopting the hierarchical softmax function, the proposed tracker can estimate infrequent classes as well as the frequent classes.

**Figure 6:**
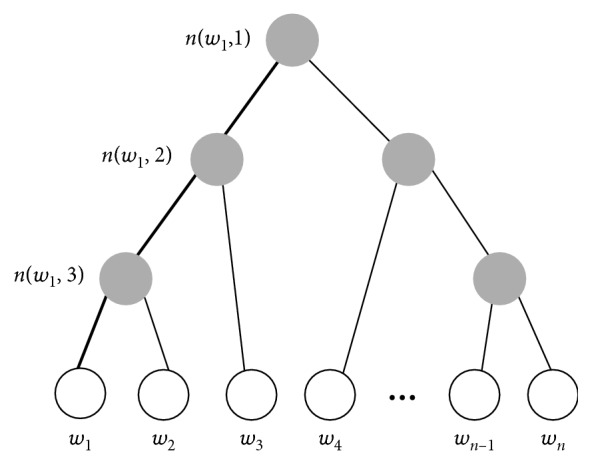
An example of hierarchical binary tree.

In the proposed model, the slots and their attributes are considered as words, and thus they are represented as a binary tree in which a word is a leaf node. Since there are *V* words, the tree has *V* − 1 inner nodes. For each leaf node, there exists only one path from a root to the leaf node that is used to estimate the probability of the word. For example, Figure 6 is a hierarchical binary tree in which the leaf nodes represent words (slot-attribute pairs) and inner nodes represent probability mass. The highlighted nodes and edges make a path from root to an leaf node *w*_1_, and *n*(*w*, *j*) means the *j*-th node on the path from root to a leaf node *w*. In the hierarchical softmax model, each of the *V* − 1 inner node has an output vector *v*′_*n*(*w*, *j*)_. The probability of a word being an output word is

(10) $$\begin{array}{c} p\left(w=w_{O}\right)=\overset{L\left(w\right)-1}{\underset{j=1}{\prod }}\sigma \left(I\left(t\right)\cdot v_{n\left(w,j\right)}^{\prime }\cdot h\right), \end{array}$$

where **I**(*t*) is an indicator function whose value is 1 if the variable *t* is true and −1 otherwise. In this equation, *t* checks if *n*(*w*, *j*+1)=lch(*n*(*w*, *j*)), lch(*n*) is the left child of node *n*, and *L*(*w*) is the length of the path. Thus, **I**(*t*) examines whether the (*j*+1) − th inner node on the path from root to *w* is equal to the left child of the *j* − th inner node on the path or not.

## 5. Experiments

### 5.1. Data Set

To examine the effectiveness of the proposed model, we used TourSG data set (http://www.colips.org/workshop/dstc4/data.html) that was used for DSTC4.  Table 1 shows the simple statistics on the data set. This data set has totally 35 dialog sessions on Singapore tour information collected from Skype call logs. Three tour guides and 35 tourists have participated in these call logs. The 35 dialog sessions are composed of 31,304 utterances and 273,580 words, respectively. The training set has seven dialogs from tour guide SG1 and SG2, the development set has three dialogs from the same guides, and the test set has three dialogs from all tour guides. Thus, the dialogs from tour guide SG3 are used only for test. As a result, this data set can be used to evaluate the robustness of dialog state trackers.

**Figure 7:**
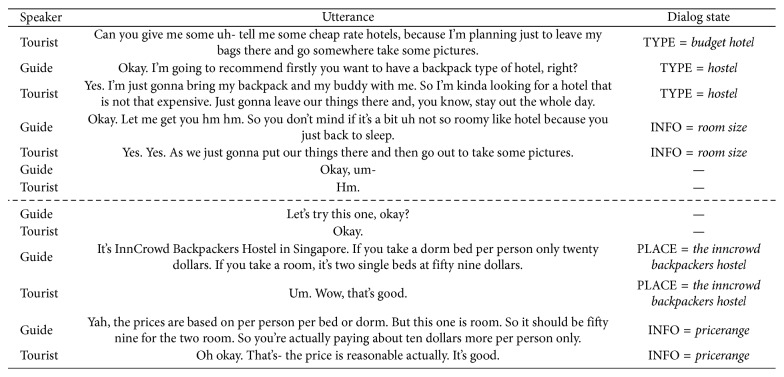
A sample dialog from TourSG data set for a topic ACCOMMODATION.

Every dialog in the data set is expressed as a series of utterances, and each utterance is an unrefined transcription containing some disfluencies such as “ah,” “uh,” or “um.” A dialog can be divided again into subdialog segments that belong to one of nine topic categories of *OPENING*, *CLOSING*, *FOOD*, *ITINERARY*, *ACCOMMODATION*, *ATTRACTION*, *SHOPPING*, *TRANSPORTATION*, and *OTHER*. The example dialog in Figure 7 has two subdialogs where a dashed line is the border between the subdialogs. As shown in “Dialog State” column of this table, not all utterances have a slot and attributes.

The topics of *OPENING*, *CLOSING*, *ITINERARY*, and *OTHER* deliver no information about dialog states. Thus, they are all merged into a single topic *OTHERS* for the informativeness classification. As a result, for the informative classifier, the five topics of *FOOD*, *ACCOMMODATION*, *ATTRACTION*, *SHOPPING*, and *TRANSPORTATION* are considered as a informative class, while the topic *OTHERS* is regarded as a noninformative class.  Table 2 summarizes the class distribution of the TourSG data set for the informativeness classifier. The training set has 9,974 informative utterances and 2,785 noninformative utterances. The development set has 4,139 informative and 673 noninformative utterances, while the test data set has 6,528 and 1,320 utterances for informative and noninformative classes, respectively.

The data set has two kinds of slots for dialog state tracking: a regular slot and an INFO slot. The regular slot is predefined to deliver the essential content of a subdialog for a specific topic. A topic contains three to seven regular slots on average. On the other hand, the INFO slot is for indicating the content mentioned implicitly in the subdialog. In the second subdialog of Figure 7, there are two slots of “PLACE” and “INFO” where “PLACE” is a regular slot and “INFO” is the INFO slot. Since the hotel name is described explicitly with the words “*InnCrowd Backpackers Hotel*,” it becomes the attribute of the slot “PLACE.” In addition, the dialog participants are discussing the price of the hotel. Thus, the attribute of the INFO slot becomes *Pricerange*, even though the words “price range” do not appear explicitly in the dialog. The possible slot types and the list of their candidate attributes vary according to a topic category, and they are described as an ontology provided by DSTC [26].

Table 3 shows the number of slots and their possible attributes for every topic category. *ACCOMMODATION*, *ATTRACTION*, *FOOD*, and *SHOPPING* contain common regular slots that refer to the names of places, the types of places, and the information of geographic areas. In detail, the topic *ACCOMMODATION* has three common regular slots, and the topic *ATTRACTION* includes five regular slots about the touristic activities, visit-time for attraction, and three common regular slots. The topic *FOOD* has seven slots that stand for the types of cuisine, the names of dishes and drinks, the time for eating, and three common regular slots. The topic *SHOPPING* has three common regular slots and time information for shopping. The last topic, *TRANSPORTATION* includes the types of transportation, the destinations and origins, the information on train lines and stations in Singapore, and the types of tickets. The regular slots have 570, 661, 1,411, 651, and 2,450 attributes in *ACCOMMODATION*, *ATTRACTION*, *FOOD*, *SHOPPING*, and *TRANSPORTATION*, respectively. On average, one regular slot has roughly 190, 122, 202, 163, and 490 attributes for each topic category. On the other hand, INFO slot has only one slot. This is because INFO is both a slot type and a slot name. It has 32, 31, 16, 16, and 15 attributes for five topic categories.

### 5.2. Evaluation Metrics and Experimental Settings

The accuracy and the F1-score are used to evaluate both the informativeness classifier and the neural dialog state tracker where they are defined as follows:

- Accuracy: the fraction of predictions in which the model output is same with the gold label
- Precision/Recall/F1
  - Precision: the fraction of predictions in the model outputs that are correctly predicted
  - Recall: the fraction of predictions in the gold labels that are correctly predicted
  - F1: the harmonic mean of precision and recall

For the informativeness classifier, the accuracy checks how correctly a classifier determines whether a given utterance is informative or not, and the F1-score shows how precise the informativeness classifier is, as well as how robust it is. In the dialog state tracking, the predictions are slot-attribute pairs. Thus, the accuracy checks how equivalent the tracker's outputs are to gold standard labels at the whole frame-level, while the precision and the recall aim to check the partial correctness at the slot-attribute level.

The parameters of the informativeness classifier and the neural dialog tracker are described in Tables 4 and 5, respectively. Every word of the utterances, the input for both the informativeness classifier and the neural tracker, is embedded into a 500-dimension vector. Three different sizes of filters are used for the informativeness classifier and the number of filters for each filter size is 128. The dropout rate of the classifier is 0.5, the batch size is 50, and the number of training epochs is 10. For the neural tracker, the hidden layer size is 500, the dropout ratio is 0.1, the learning rate is 0.005, and the number of epochs is 100,000.

### 5.3. Experimental Results

Table 6 shows the classification performance of the informativeness classifier. The classifier achieves the accuracy of 93.16% and F1-score of 93.17 for test set, while the classifier shows a relatively low precision of 90.89%. The reason for the relatively low precision is that there are some utterances about money in the noninformative class. For instance, the classifier predicts an utterance “Okay, maybe a thousand dollars for a day.” as informative due to the words “thousand dollars” and “a day,” but its gold standard label is noninformative. Since the dialog to which the utterance belongs is an opening section before recommending tour information, the utterance is a noninformative utterance for tracking dialog. This problem occurs because the classifier determines the informativeness of utterances without any contextual information of a dialog flow.

To show the feasibility of the proposed model, the dialog state tracking performance of the proposed model is compared with those of the participants in the DSTC4 challenge. The string match tracker determines the slot and the attribute by a simple exact string match between the slot and the given utterance [26]. The LSTM-based neural tracker proposed by Yoshino et al. [20] is a pure LSTM without the attention mechanism. Thus, it can be directly compared with the proposed model. Tables 7 and 8 show the performance of the trackers. The proposed model outperforms its competitors in both accuracy and F1-score. Especially, it achieves four times higher accuracy than the LSTM which is a neural network-based model and is a similar approach as our proposed model. This result means that handling necessary information from utterance is important to tracking dialog state. The main difference between the proposed model and the LSTM is the presence of the attention mechanism. Thus, it can be inferred that it is important in dialog state tracking to focus on some salient words while processing a user utterance.

**Figure 8:**
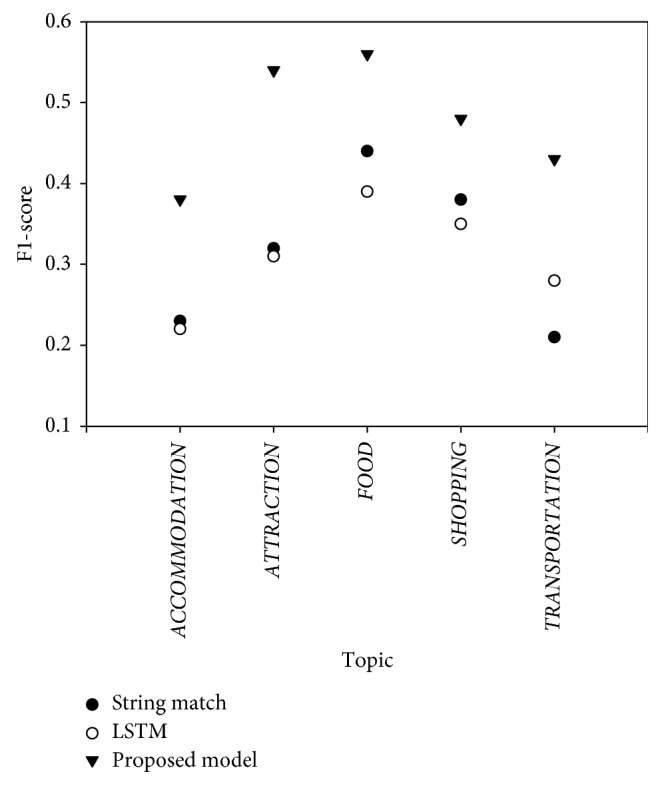
The F1-score of the proposed model per topic.

The performance of the proposed model per topic is investigated in Figure 8. The performance is measured for five topics related with dialog state tracking. The proposed model achieves the best performance at every topic. Especially, the proposed model only obtains lower performance at the topic *ACCOMMODATION*. The reason why the proposed model shows poor performance for *ACCOMMODATION* is that there are many utterances mentioning some locations in the dialogs of *ACCOMMODATION*, but they are often related with other topics such as *TRANSPORTATION* and *SHOPPING*. For instance, for the utterance “ten minutes walk to the mrt train station,” the proposed model predicts its slot as “STATION” and its attribute as “MRT train station.” This prediction seems to be relevant since a train station is usually related to *TRANSPORTATION*. However, the dialog is about the places near the suggested accommodation. Thus, the gold standard slot for the utterance is “NEIGHBORHOOD,” and the gold standard attribute is “Kampong Glam,” a location name.

Table 9 implies the dialog state tracking accuracy of the proposed model for every slot in a topic category. Note that each accuracy is computed at the utterance level, not the subdialog level. The regular slots which have distinguishable attributes like “TIME” and “TYPE_OF_PLACE” show moderately high accuracy, while some regular slots such as “NEIGHBOURHOOD,” “PLACE,” or “DISH” achieve low accuracy. The reason why the proposed model shows poor performance for some regular slots is that they have specific names for their attributes such as restaurant name, street name, and dish name that are extremely infrequent in data set. The proposed model also achieves meaningful performance for INFO slot. The high performance for INFO slot implies that the proposed model can catch the contents mentioned implicitly in an utterance.

To examine the effectiveness of the attention mechanism and the hierarchical softmax, three variations of the proposed model are compared with the proposed model. One variant (NT-Atten-NoHier) is the neural tracker with the attention mechanism and the general softmax. This is designed to inspect the effectiveness of the attention mechanism in dialog state tracking. Another (NT-NoAtten-Hier) is the neural tracker without the attention but with the hierarchical softmax. This is prepared to examine the hierarchical softmax. The third (NT-Atten-Hier) is the proposed model, the neural tracker with both the attention and the hierarchical softmax. The informativeness classifier is not used in comparing them.

Tables 10 and 11 prove the effectiveness of the attention mechanism and the hierarchical softmax. The proposed model (NT-Atten-Hier) outperforms both NT-Atten-NoHier and NT-NoAtten-Hier, which implies that the attention mechanism and the hierarchical softmax are all helpful in enhancing the neural tracker. One thing to note in these tables is that the performance improvement of the neural tracker is made mainly by the attention mechanism. NT-Atten-Hier achieves 1.6 times higher accuracy than NT-NoAtten-Hier, but it shows a similar accuracy with NT-Atten-NoHier. Nevertheless, it is also true that the hierarchical softmax enhances the neural tracker. This is proved by the fact that the performance of NT-Atten-Hier is slightly higher than that of NT-Atten-NoHier.

Table 12 shows the effectiveness of the informativeness classifier. The F1-score of the proposed pure neural tracker, NT-Atten-Hier, is 0.3977, but it goes up to 0.4174 when the informativeness classifier is applied in front of the neural tracker as in Figure 3. The improvement by the informativeness classifier is also discovered in accuracy. This is because the adoption of the informativeness classifier reduces the possibility of misjudgments by noninformative utterances.

To analyze the shortcomings of various neural trackers, we use three types of slot-level errors that were defined in the DSTC challenge. The error types are as follows:

- Missing attribute: the actual dialog has the attributes for a specific slot, but the tracker does not output any attribute for the slot
- Extraneous attribute: no attribute for a slot is contained in an actual dialog, but the tracker outputs some attributes for the slot
- False attribute: the attribute for a slot is specified in an actual dialog, but the tracker outputs a different attribute from the dialog

According to the result of DSTC4 challenge [26], the largest error type is the missing attribute.

**Figure 9:**
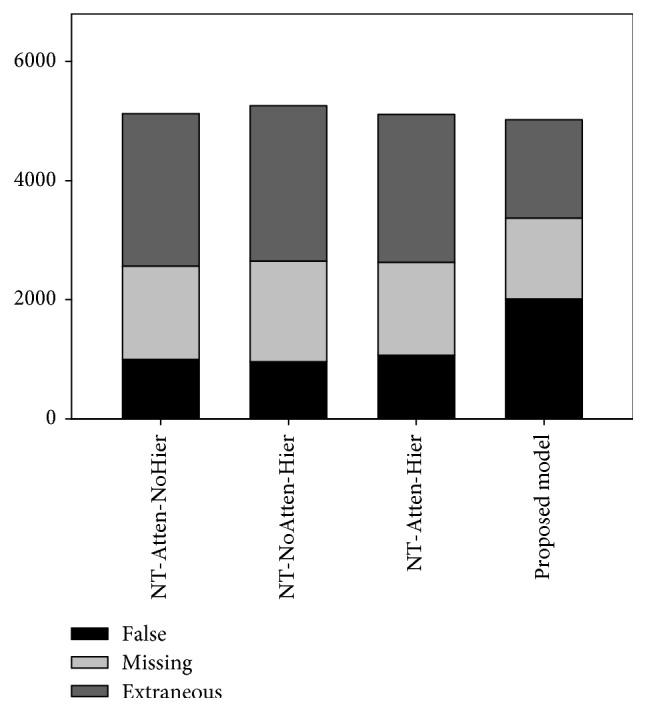
The number of errors according to error types.

Figure 9 depicts the number of errors in each error type by the variants of the proposed model. In this figure, “Proposed Model” implies that NT-Atten-Hier with the informativeness classifier. The number of errors in missing attribute and extraneous attribute is much reduced in “Proposed Model” while sacrificing somewhat of false attribute. Therefore, the informativeness classifier is proved to be helpful actually in reducing the errors in missing and extraneous attributes. Many errors in false attribute are made by a limited context of a given utterance. For instance, let us consider an utterance “uh another place you might want to check out is opposite to Sentosa island and it is called Vivocity.” In this utterance, a user wants to know another shopping place. Since the proposed model focuses only on the utterance, it predicts the slot for the utterance as “NEIGHBOURHOOD” and its attribute as “Sentosa.” This is wrong but plausible predictions, since the utterance also contains locational information semantically. That is, many errors by the proposed model are not critical in understanding the user intention.

## 6. Conclusion

This paper has proposed a task-oriented dialog state tracker in two steps. The proposed tracker is composed of an informativeness classifier and a neural dialog state tracker. It first filters noninformative utterances in dialog by using informative classifier. The informativeness classifier is implemented by CNN which showed good performance in sentence classification. In the next step, the neural tracker determines dialog states from informative utterance with attention mechanism and hierarchical softmax. By adopting the attention mechanism and hierarchical softmax, the proposed model can not only focus on salient word in a given utterance but also deal with a large of vocabulary in dialog efficiently. The experimental results on dialog state tracking to examine the capability of the proposed model show that the proposed model outperforms both the neural tracker without informativeness classifier, the attention mechanism, and the hierarchical softmax as well as the previous work, which implies the plausibility of adopting them in dialog state tracking.

There is still some room for its improvement although the proposed model achieves an acceptable result. One possible improvement for the current neural tracker is to preserve topic consistency when a model predicts slots. The proposed model considers only a given utterance when it predicts a slot. However, since a general dialog has a single topic, the topic of the utterances in the same dialog segment has to continue when a model predicts the slots of the utterances. Thus, we will consider a dialog history to catch the flow of a dialog and to preserve topic consistency of predicting slots in the future work.

## Figures and Tables

**Table 1:** A simple statistics on the TourSG data set.

Set	No. of dialogs				No. of segments							No. of utterances
	SG1	SG2	SG3	Total	Acco	Attr	Food	Shop	Trsp	Other	Total	
Training	7	7	0	14	187	762	275	149	374	357	2,104	12,759
Development	3	3	0	6	94	282	102	67	87	68	700	4,812
Test	3	3	3	9	174	616	134	49	174	186	1,333	7,848

**Table 2:** The class distribution for the informativeness classifier.

Set	Informative	Noninformative
Training	9,974	2,785
Development	4,139	673
Test	6,528	1,320

**Table 3:** The number of slots (regular, INFO) and their attributes.

Topic category	Regular slot			INFO slot	
	No. of slot	No. of attribute	No. of avg. attribute per slot	No. of slot	No. of attribute
*ACCOMMODATION*	3	570	190	1	23
*ATTRACTION*	5	611	122	1	31
*FOOD*	7	1,411	202	1	16
*SHOPPING*	4	651	163	1	16
*TRANSPORTATION*	5	2,450	490	1	15

**Table 4:** The parameters for the informativeness classifier and their values.

Parameter	Value
Embedding size	500
Filter sizes	2, 3, 4
The number of filters	128
Dropout ratio	0.5
Batch size	50
Number of epochs	1,000

**Table 5:** The parameters for the neural dialog state tracker and their values.

Parameter	Value
Embedding size	500
Hidden layer size	500
Dropout ratio	0.1
Learning rate	0.005
Number of epochs	100,000

**Table 6:** The classification performance of the informativeness classifier.

Set	Accuracy	Precision	Recall	F1-score
Development	0.9484	0.9202	0.9820	0.9501
Test	0.9316	0.9089	0.9557	0.9317

**Table 7:** The comparison of the dialog state tracking accuracies between the proposed model and DSTC4 participants.

Model	Accuracy
String match [26]	0.0374
LSTM [20]	0.0270
*Proposed model*	**0.0852**

**Table 8:** The comparison of the dialog state tracking F1-scores between the proposed model and DSTC4 participants.

Model	Precision	Recall	F1-score
String match [26]	0.3589	0.1925	0.2506
LSTM [20]	0.3400	0.2010	0.2530
*Proposed model*	**0.5173**	**0.3499**	**0.4174**

**Table 9:** The dialog state tracking accuracy of proposed model for every slot in topic category.

Topic	Slot	Accuracy
*ACCOMMODATION*	INFO	32.4
	TYPE_OF_PLACE	48.3
	PLACE	18.7
	NEIGHBOURHOOD	8.8
*ATTRACTION*	INFO	21.1
	TYPE_OF_PLACE	65.5
	ACTIVITY	55.7
	PLACE	4.6
	TIME	63.3
	NEIGHBOURHOOD	17.8
*FOOD*	INFO	37.1
	CUISINE	47.1
	TYPE_OF_PLACE	68.6
	DRINK	62.2
	PLACE	7.7
	MEAL_TIME	73.9
	DISH	5.8
	NEIGHBOURHOOD	27.3
*SHOPPING*	INFO	41.3
	TYPE_OF_PLACE	41.9
	PLACE	4.8
	NEIGHBOURHOOD	4.1
	TIME	75.0
*TRANSPORTATION*	INFO	23.9
	FROM	5.3
	TO	5.6
	STATION	14.7
	LINE	16.1
	TYPE	42.8

**Table 10:** The dialog state tracking accuracy of the proposed model's variants.

Model	Accuracy
NT-Atten-NoHier	0.0672
NT-NoAtten-Hier	0.0422
*NT-Atten-Hier*	**0.0697**

**Table 11:** The F1-score of the dialog state tracking of the proposed model's variants.

Model	Precision	Recall	F1-score
NT-Atten-NoHier	0.4403	0.3335	0.3795
NT-NoAtten-Hier	0.3316	0.3058	0.3181
*NT-Atten-Hier*	**0.4662**	**0.3469**	**0.3977**

**Table 12:** The effect of the informativeness classifier in the proposed model.

Model	Accuracy	Precision	Recall	F1-score
Without informativeness classifier	0.0697	0.4662	0.3469	0.3977
*With informativeness classifier*	**0.0852**	**0.5173**	**0.3499**	**0.4174**

## Data Availability

We used dialog state data (TourSG data set) which are provided from ETPL-A*∗*STAR (dialog state tracking challenge 4 organization). The TourSG is a research dialog corpus in the touristic domain and is divided by train/development data and test data. Train and Development data include manual transcriptions and annotations at both utterance and subdialog levels for training model and fine-tuning its parameters. Test set includes manual transcriptions for evaluating the model. All researcher/company can access the data in following site: https://www.etpl.sg/innovation-offerings/ready-to-sign-licenses/toursg-a-research-dialogue-corpus-in-the-touristic-domain. Researchers or their company need to pay a license fee for getting the TourSG data set after signing a license agreement with ETPL-A*∗*STAR. The data set include transcribed and annotated dialogs, as well as ontology objects describing the annotations.
